# Evaluating the home-based intervention strategy (HIS-UK) to reduce new chlamydia infection among young men aged 16–25 years by promoting correct and consistent condom use: findings from a randomised controlled trial

**DOI:** 10.1186/s12913-024-11911-2

**Published:** 2024-12-18

**Authors:** Nicole Stone, Cynthia Graham, Stephen Bremner, Nuala McGrath, Rowena Bedford, Katherine E Brown, Katie Newby, Amanda Clarke, Louise Jackson, Leanne Morrison, Tom Nadarzynski, Ye To

**Affiliations:** 1 Centre for Sexual Health Research, University of Southampton, Southampton, UK; 2 Kinsey Institute, Indiana University, Bloomington, USA; 3 Department of Psychology, University of Southampton, Southampton, UK; 4 Institute of Applied Health Research, College of Medical and Dental Sciences, University of Birmingham, Birmingham, UK; 5 University Hospitals Sussex NHS Foundation Trust, Brighton, UK; 6 Department of Primary Care and Public Health, Brighton & Sussex Medical School, University of Sussex, Brighton, UK; 7 CHERISH programme, School of Primary Care, Population Sciences and Medical Education, University of Southampton, Southampton, UK; 8 Department of Social Statistics & Demography, University of Southampton, Southampton, UK; 9 Africa Health Research Institute, KwaZulu-Natal, South Africa; 10 Public Health and Applied Behaviour Change Laboratory, University of Hertfordshire, Hatfield, UK; 11 Primary Care Research Centre, University of Southampton, Southampton, UK; 12 School of Social Sciences, University of Westminster, London, UK; 13 Brighton and Sussex Clinical Trials Unit, University of Sussex, Brighton, UK; 14 NDORMS, Medical Sciences Division, University of Oxford, Oxford, UK

**Keywords:** Chlamydia, Sexually transmitted infection, STI, Condom, Intervention, Lubricant, Young men, Behaviour change

## Abstract

**Background:**

Correct and consistent condom use is the most effective method to reduce transmission of sexually transmitted infections (STIs).

**Objective:**

To compare the HIS-UK intervention to usual condom information and distribution care for effect on chlamydia test positivity.

**Methods:**

*Trial design* A 3-parallel arm randomised controlled trial (1:1:1 allocation, two intervention arms vs. control). Randomisation using permuted blocks of varying lengths, with stratification by site, ethnicity and sexual-partnering risk. Repeated measures design with monthly follow-up to six months post-randomisation.

*Setting* Sexual health services in seven NHS Trusts and one university medical centre. Telephone and video consultations, online and in participants’ homes in England, UK.

*Participants* Target sample of 2231 men and people with penises, aged 16-25, at risk of STIs.

*Intervention* HIS-UK delivered (1) face-to-face by health professionals (proHIS) or (2) digitally (eHIS). Two-weeks self-practice and experimentation using the HIS-UK condom kit.

*Primary health outcome* Chlamydia test positivity by six-months.

*Secondary outcomes* Frequency of unprotected sexual intercourse, reported condom use errors and problems, attitudes and use experience.

*Analyses* Chlamydia test positivity by six months analysed by logistic regression. Secondary outcomes analysed using linear mixed effects models with fixed effects and a random effect for the repeated measures, and generalised estimating equations with a logit link, adjusting for fixed effects and specifying an autoregressive-1 correlation structure.

**Results:**

Seven hundred twenty-five participants (proHIS:241, eHIS:243, control:241) randomised. 575 participants completed all baseline activities, 189 (32.9%) reached six-months post-randomisation. The absolute difference in chlamydia test positivity between arms was -4.9 percentage points at six months (7.9% HIS-UK, 12.8% control). The odds of chlamydia test positivity during follow-up were 55% lower for HIS-UK participants (*p*=.261).

HIS-UK showed a positive impact on recent condom use over time (*p*<.001). Significant reductions in condom errors and problems among HIS-UK participants were observed (*p*=.035). Lubricant use increased among HIS-UK participants, with evidence of an intervention-by-time interaction (*p*=.051), and a decline in poor condom fit and feel reports, but without intervention effect.

**Conclusions:**

This study provides valuable insights into the potential of HIS-UK to enhance sexual health practices among at-risk populations at-risk of STI transmission.

**Trial registration:**

ISRCTN registration: 11400820 (23/10/2019).

**Supplementary Information:**

The online version contains supplementary material available at 10.1186/s12913-024-11911-2.

## Introduction

### Background

The Department of Health and Social Care recommends the use of evidence-based preventative interventions to decrease rates of sexually transmitted infections (STIs) [1]. However, national guidance on behavioural interventions for STI prevention remains limited. A comprehensive review of evidence on safer sex advice recommended the incorporation of brief behaviour change interventions into routine care for those at elevated risk of STIs, with targeted interventions emphasising skills acquisition, communication competencies, and motivation to adopt safer sexual behaviours [2–4].

Guidelines from the National Institute for Health and Care Excellence (NICE) further emphasise teaching young people effective and safe condom use for the prevention of pregnancy and STIs through education, information, and demonstrations before the distribution of a variety of condom and lubricant supplies. NICE also recommends research to identify the most effective behaviour change techniques for supporting consistent and correct condom use post-distribution [5–7].

Many condom negotiation and motivational interventions are resource intensive. With a significant reduction in funding for sexual health services, there is a growing need for innovative approaches to STI prevention that minimise staff time and clinic attendance for the delivery of routine care [8]. Digital behaviour change interventions (DBCIs) emerge as a promising solution, whilst also removing accessibility barriers which contribute to health inequalities. DBCIs enable participation at users’ convenience, provide anonymity, and alleviate fears of stigmatisation. They also offer the added advantage of delivering consistent and standardised information, with implementation costs typically lower than for traditional methods [9]. Although condom education is included in school-based sex education in the U.K., in recognition of the increasing use of the internet for accessing health information, National Health Service (NHS) England underscores the importance of harnessing digital technologies in all aspects of healthcare delivery [10].

Systematic reviews evaluating the efficacy of in-person and DBCIs for consistent condom use have yielded mixed results [11–14]. Challenges include poor-quality trials and a failure to accurately identify or describe the active components or behaviour change techniques, thus limiting the ability of studies to guide future intervention development. However, emerging evidence suggests that brief behavioural interventions incorporating identifiable and evidence-based components can be used to effectively reduce STI acquisition [3, 15–20].

Development of the HIS-UK intervention was guided by the Information-Motivation-Behavioural Skills (IMBS) theoretical model of behaviour change which has been shown to be effective with young people [21, 22]. In HIS-UK the impetus for change comes from the promotion of solitary experimentation with different condom and lubricant types and a focus on pleasure [23–26].

### Objectives

The HIS-UK trial was designed to test whether the HIS-UK intervention, delivered by two models – face-to-face (proHIS) and digitally (eHIS) – was effective in promoting behaviour change and reducing chlamydia test positivity among men aged 16–25 years compared to usual condom information and distribution care offered by the NHS and more widely.

The secondary objectives, through which the primary objective would be realised, included the enhancement of condom use attitudes and experiences and improvements in correct and consistent condom use.

### Impact of COVID-19

The HIS-UK trial opened to recruitment in March 2020. On March 30th, recruitment was halted in line with National Institute for Health and Social Care Research (NIHR) guidance due to the COVID-19 pandemic. In June 2021, recruitment resumed with a revised protocol reflecting post-COVID NHS working directives; specifically, a reduction in direct clinical contact time with trial participants, and increased provision of remote research participation and care delivery. Further protocol amendments expanded community-based recruitment opportunities to increase recruitment within the remaining study timeframe (for details see [27]). This article presents the methodology as published in the final approved protocol.

## Methodology

### Trial design

A 3-parallel arm randomised controlled superiority trial (1:1:1 allocation, two intervention arms vs. control), compared the effectiveness of HIS-UK to usual condom information and distribution care. A repeated measures design with baseline measurement, monthly follow-up questionnaires and up to three STI screening points was employed. The trial adhered to CONSORT guidelines.

### Participants

#### Clinical

Participants were recruited from clinical trial recruitment sites (CTRS) in England, including integrated sexual health and Genitourinary Medicine (GUM) clinics located in seven NHS Trusts and one university health centre. Trained NHS staff at CTRS directly approached potential participants with information about the study and were responsible for the delivery of the research procedures.

#### Community

GP practices and sexual health services located in 13 NIHR Clinical Research Network areas additionally acted as participant identification centres (PICs), sending text message adverts to potential participants. Community outreach channels and social media platforms also promoted the trial using targeted adverts, signposting to the study website for online eligibility screening and self-registration. Researchers at the University of Southampton handled the research delivery to self-referral participants outside CTRS catchment areas.

#### Eligibility

Anyone with a penis was eligible if they were aged between 16 and 25 years, UK residents and at risk of STIs through reporting of condom use errors (breakage/slippage) or condomless intercourse (anal/vaginal) with a casual or new sexual partner in the past three months. Exclusions included recognised latex allergy, no internet access, and limited English proficiency hindering understanding of trial instructions.

### Intervention

The HIS-UK intervention comprised three elements: (1) Focused condom and lubricant education and training, (2) Solitary condom and lubricant experimentation, and (3) Online ratings of condoms and lubricants.

The HIS-UK condom and lubricant education and training session was delivered either face-to-face by a trained professional (proHIS) or online via an interactive digital media website (eHIS). The content was devised as an extension of the usual information and condom demonstration practiced by health promotion professionals and offered to young people in condom distribution settings. The session was designed to be delivered within 15 min. Topics covered included correct condom use, maximising pleasure, finding the perfect condom fit, condom types, the benefits of lubricants and how and where to apply.

HIS-UK participants were additionally provided with a condom kit containing 12 sachets of lubricant of three different types and 24 condoms of 12 types (variation by shape, size, material, texture and thickness) and commenced a two-week experimentation and self-practice period to complete at home. Following guided instructions, participants practiced applying, using (masturbating with) and removing the different condoms in “low pressure” situations (i.e., not in the presence of a sexual partner). As participants tried out each condom and lubricant, they were asked to consider ‘fit & feel’ and focus on pleasurable sensations in order to build positive associations between condom use and sexual enjoyment. After experimentation with each condom and lubricant, participants were prompted to complete an online rating and evaluation form.

Control arm clinical participants received a usual condom information and distribution care consultation. Community participants in the control arm were asked to review selected online safe sex advice and condom information published by NHS, Governmental and charitable organisations.

### Outcomes

Chlamydia positivity, the primary outcome, was measured at baseline and six months post randomisation through bio-marker testing. Participants who tested positive were offered treatment. Additionally, monthly self-reported STI diagnosis data were collected via online questionnaires (T1-T6). Secondary process indicators, assessed using validated questionnaires and a sexual behaviour and contraceptive use survey, were measured at baseline and repeated monthly by online self-completion questionnaire.

#### Scales and scores

##### Condom use experience

Condom use experience was assessed using a seven-item subscale of the validated Condom Barriers Scale [28], measured on a 5-point Likert scale (strongly disagree to strongly agree). Example items include: *Condoms don’t fit properly* and *Condoms rub and cause irritation*.

##### Condom use attitudes

The Condom Use Attitudes Scale was adapted from the Multidimensional Condom Attitude Scale [23, 29]. Example items included: *Condoms ruin intercourse* and *Condoms interrupt the mood*.

##### Pleasurable condom use

The 5-item pleasurable condom use scale was created using findings from the HIS-UK feasibility study [24]. Example items included: *Condoms can add excitement to foreplay*, and *Condoms can help you have better sex*.

##### Condom use self-efficacy

Condom use self-efficacy, assessed by the established 7-item Condom Use Self-Efficacy Scale [30], asked participants how difficult or easy they would find certain behaviours, for example, *Apply condoms correctly*, and *Keep an erection when using a condom*. A second 3-item measure assessed confidence in using condoms effectively with partners and included items such as: *I feel confident putting on a condom in front of my partner*.

##### Condom use errors and problems

The condom use errors and problems score was created from the Condom Use Errors and Problems Survey [31]. Example items included: *Application of the condom the wrong way up*,* Losing an erection whilst applying a condom*, *Not using a condom from the start to finish of intercourse* and *Breakage of the condom during intercourse*. Participants reported whether any of the statement items had occurred or been experienced at last condom use (Yes/No). Responses were summed to give a final score, with a higher score reflective of more reported errors and problems.

##### Condom use fit and feel

Four items from the Condom Fit and Feel Scale [32] explored men’s general experiences with condom fit and feel. Responses were provided on a frequency Likert scale (1-never applies to me to 4-always applies to me). Example statement items included: *Condoms are too short for my penis* and *Condoms are too tight for my penis*. A second binary measure of any reported fit and feel issues at last condom use (yes/no) was also used.

##### Condom use motivation

To measure condom use motivation, participants indicated their level of agreement on a five-point Likert scale (strongly disagree to strongly agree) with the statement: *I want to use condoms with my partner(s)*.

### Sample size

The planned analysis of HIS-UK, assessing the effectiveness of proHIS and eHIS in reducing chlamydia test positivity, employed an overall Type I error rate of 5%. The trial was designed to detect a reduction in positivity rates from 11% (observed among men aged 15–24 years in national screening [33]) to 6% at six months post randomisation. Based on pilot data suggesting equal intervention effectiveness across subgroups (deprivation, ethnicity, sexual orientation, age) and aiming for 85% power and assuming 36% attrition (observed in feasibility testing), the trial targeted 2231 participants across all arms (G*Power 3.1.9.2) [24, 25, 34]. Progression criteria were established for recruitment, uptake of STI screening and follow-up attrition and the trial protocol was published [27].

### Randomisation

The trial was administered using *Lifeguide*, an interactive web-based intervention software platform and secure validated data management system. *Lifeguide* was programmed to automate and deliver all trial procedures, collect all participant data and manage participant work-flow and communications. Trial arm allocation was actioned by an in-built algorithm generating permuted blocks of randomly varying length to preserve concealment and maintain balance, with stratification by site, ethnicity and sexual partnering risk. Participants were allocated at a ratio of 1:1:1. Neither participants nor staff using *Lifeguide* were blinded due to the nature of the intervention.

### Impact of COVID-19

Regrettably, the trial funder did not extend the study timeline to cover time lost due to COVID-19 and the imposed halt to recruitment. The HIS-UK trial was consequently unable to recruit to the target sample size within the reduced timeframe. Considering the participant shortfall, the statistical analysis plan was amended. The proHIS and eHIS participants were combined to form a single HIS-UK trial arm for comparative purposes and the extended impact analysis (post 6 months follow-up) cancelled.

### Analysis

Chlamydia test positivity was analysed descriptively by trial arm. Additionally, a logistic regression model was fitted to assess intervention effect at follow-up after adjusting for chlamydia test result at baseline and the stratification factors of recruitment (site, ethnicity and sexual partnering risk).

Multiple imputation methodology was used as required for all user-missing secondary outcome measures among eligible participants in each analysis. Furthermore, mixed models were fitted to handle unbalanced data due to unequal repeats of monthly outcome measures among eligible participants in each secondary analysis (system-missing due to N/As, i.e. not using a condom during a specified month).

The estimated intervention effects between HIS-UK and Control were calculated (e.g. difference in estimated marginal means or adjusted odds ratios, as applicable), along with the corresponding 95% confidence intervals and p-values from tests of no intervention effect.

For continuous secondary outcomes, linear mixed effects models were used, incorporating the fixed covariates of time (questionnaire month), intervention group (HIS-UK/Control), and the stratification factors. A time by intervention group interaction term was also tested. Repeated measurements were addressed through a random effect. Binary outcomes were analysed using logistic regression models fitted by generalised estimating equations with an autoregressive-1 correlation structure. In all models, time was treated as an ordered categorical variable in acknowledgment of the uneven measurement intervals for individual participants and the delivery of the intervention between two time points (Baseline-T1).

All analyses were performed using IBM SPSS Statistics version 29.0.1.

## Results

**Fig. 1:**
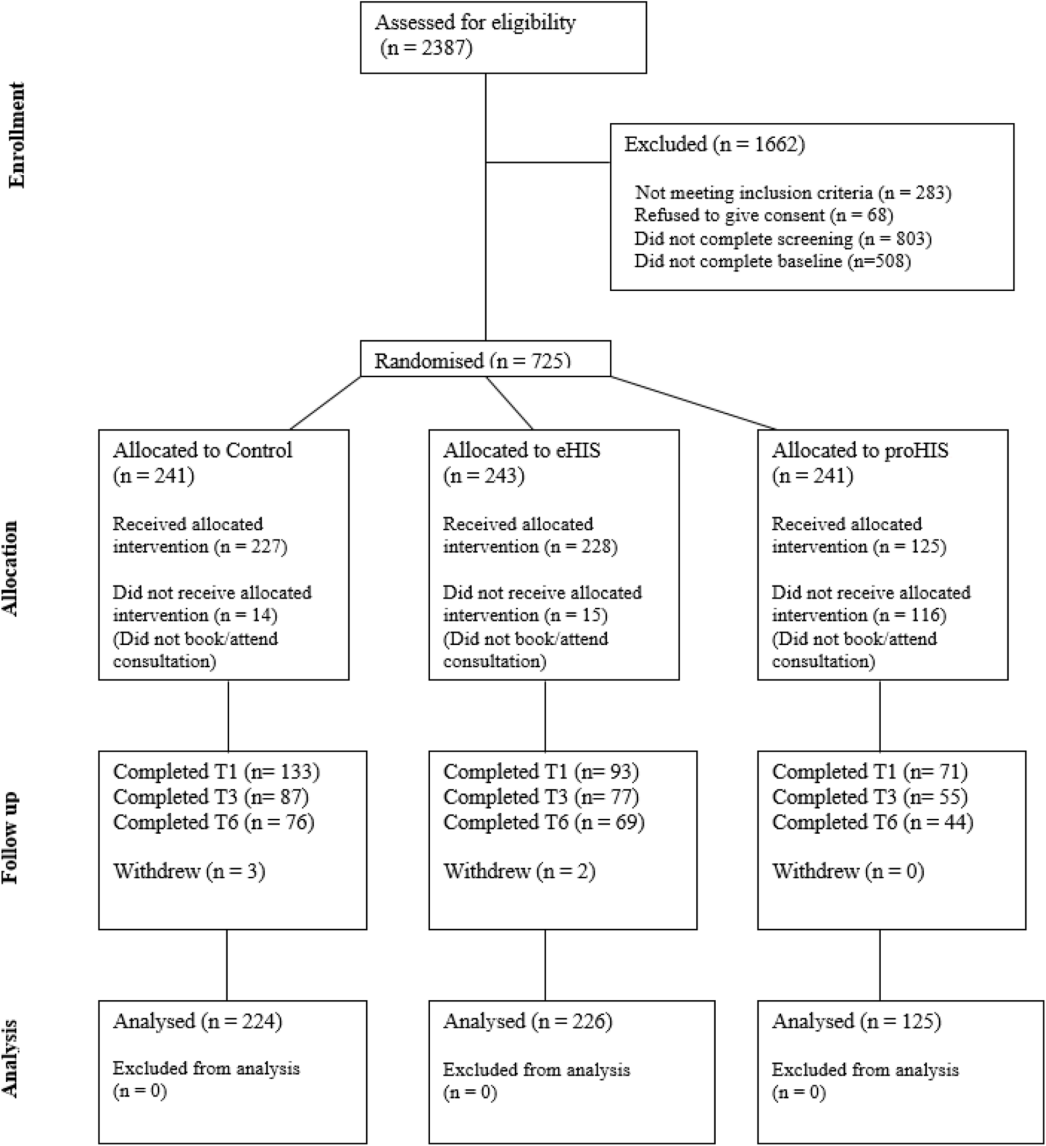
CONSORT Diagram

Between June 2021 and February 2023, 8528 men accessed the trial website and 2387 (28.0%) consented to eligibility screening. In total, 1233 (51.7%) eligible participants were identified and invited to register for the trial (see Fig. 1). Of those, 725 (58.8%) accepted the invitation, registered and were randomised to one of the three trial arms (proHIS: 241, eHIS: 243, Control: 241), 32.5% of the target sample. Five hundred and eighty (80%) participants completed all baseline activities and received condom care as per randomisation (proHIS: 125 (51.9%), eHIS: 228 (93.8%), Control: 227 (94.2%)). Two hundred and ten participants were linked with a CTRS (36.2%), and 370 (63.8%) were community participants.

The final cohort eligible for analysis consisted of 575 participants due to participant withdrawal and associated requests for the deletion of any data held (*n* = 5).

### Baseline characteristics

The median age of fully recruited participants at randomisation (*N* = 575) was 21.0 years. The majority identified as White (81%) and participants were evenly distributed across social deprivation quintiles (Index of Multiple Deprivation ascribed by postcode [35]), see Table 1.

**Table 1:** Descriptive statistics of demographic and background characteristics by arm and for total sample

Variable	HIS-UK (*n* = 351)	Control (*n* = 224)	Total (*N* = 575)
Median age	21 IQR: 20,23	22 IQR: 20,24	21 IQR: 20,24
**Ethnicity**			
White	284 (80.9%)	183 (81.7%)	467 (81.2%)
Black	21 (6.0%)	13 (5.8%)	34 (5.9%)
South Asian	26 (7.4%)	15 (6.7%)	41 (7.1%)
Other	20 (5.7%)	13 (5.8%)	33 (5.7%)
**Education and training**^**a**^			
Full-time	175 (49.9%)	97 (43.3)	272 (47.3%)
Part-time	22 (6.3%)	16 (7.1%)	38 (6.6%)
No	152 (43.3%)	111 (49.6%)	263 (45.7%)
**Employment**			
Full-time	149 (42.5%)	100 (44.6%)	249 (43.3%)
Part-time	93 (26.5%)	62 (27.7%)	155 (27.1%)
No	106 (30.2%)	62 (27.7%)	168 (29.2%)
**Social Deprivation**			
1-Most deprived	57 (16.2%)	39 (17.4%)	96 (16.7%)
2	76 (21.7%)	53 (23.7%)	129 (22.4%)
3	85 (24.2%)	41 (18.3%)	126 (21.9%)
4	77 (21.9%)	41 (18.3%)	118 (20.5%)
5-Least deprived	44 (12.5%)	33 (14.7%)	77 (13.4%)
**Highest Level of Education Completed**			
GCSE / BTEC Level 1–2	46 (13.1%)	34 (15.2%)	80 (13.9%)
A-Level / BTEC Level 3	168 (47.9%)	97 (43.3%)	265 (46.1%)
BTEC Level 4–7	11 (3.1%)	11 (4.9%)	22 (3.8%)
Degree	94 (26.8%)	55 (24.6%)	149 (25.9%)
Masters	25 (7.1%)	18 (8.0%)	43 (7.5%)
Doctorate	0 (0.0%)	2 (0.9%)	2 (0.3%)
Other	6 (1.7%)	6 (2.7%)	12 (2.1%)

^a^Education and training includes both education at schools/colleges/universities but also vocational and technical colleges

The average number of lifetime sexual partners was 10, 93% (*n* = 537) of participants had ever used a condom, and 30% (*n* = 170) had previously received a positive STI diagnosis. At baseline, 495 (86%) participants reported being sexually active during the previous four weeks. Of those who were sexually active, 41% reported use of a condom on at least one occasion, see Table 2.

**Table 2:** Baseline descriptive statistics of sexual behaviour in last 4 weeks by arm and for total sample

Variable	HIS-UK *n* = 310	Control *n* = 185	Total *N* = 495
Number of episodes of intercourse (median)	5, IQR: 2,10	4, IQR: 2,8.5	4, IQR: 2,10
Intercourse with a			
Man	90 (29.0%)	49 (26.5%)	139 (28.1%)
Woman	226 (72.9%)	137 (74.1%)	363 (73.3%)
Non-binary individual	5 (1.6%)	4 (2.2%)	9 (1.8%)
Intercourse with a			
Regular/long term partner	150 (48.4%)	83 (44.9%)	233 (47.1%)
New/casual partner	223 (71.9%)	145 (78.4%)	368 (74.3%)
Condom use	118 (38.1%)	84 (45.4%)	202 (40.8%)
Of those with lubricant	62 (52.5%)	40 (47.6%)	102 (50.5%)

### Primary health outcome

#### Chlamydia test positivity

At six months the absolute difference in chlamydia test positivity between HIS-UK and the Control was −4.9% points (95% CI: −15.7, 5.9); 7.9% for HIS-UK participants and 12.8% for the control participants (Table 3). After accounting for baseline chlamydia screening, ethnicity, recruitment site and sexual risk, the odds of receiving a positive test at follow-up were 55% lower for HIS-UK participants as compared to the control participants (reference category) (adjusted odds ratio (AOR): 0.45 (95% CI: 0.113, 1.805), *p* = .261).

**Table 3:** Descriptive statistics and intervention parameter estimates (adjusted odds ratios) of chlamydia test positivity at baseline and by 6-months post-randomisation, by trial arm and for total sample

Chlamydia test positivity	HIS-UK	Control	Between Arm Absolute Difference *p*.*p*.^a^ (95% CI)	AOR^b^ (95% CI)	Total
Baseline	*n* = 199 17 (8.54%)	*n* = 113 13 (11.50%)			*n* = 312 30 (9.62%)
By 6 months (T6)	*n* = 76 6 (7.89%)	*n* = 47 6 (12.77%)	− 4.87 (−15.66, 5.92)	0.45 (0.113, 1.805) *p* = .261	*n* = 123 12 (9.75%)

^a^*p.p*. percentage point, ^b^Logistic regression model: Trial group (control ref), Chlamydia test positivity at baseline, Ethnicity, Site, Sexual risk

### Secondary outcomes

The secondary process outcomes reflected condom competency and enjoyment. Indicators modelled included the condom attitudes and opinion scales items, behavioural measures of lubricant and condom use (condom use during last month, condom use at last intercourse, and consistency of condom use during the previous four weeks).

Attitude and opinion scale data were collected monthly from all participants; as such all 575 participants were included in the analyses, see Table 4. Behavioural measures were collected monthly from eligible participants only and the numbers included in each analysis are displayed in Tables 5 and 6.

#### Condom attitudes and opinions

**Fig. 2:**
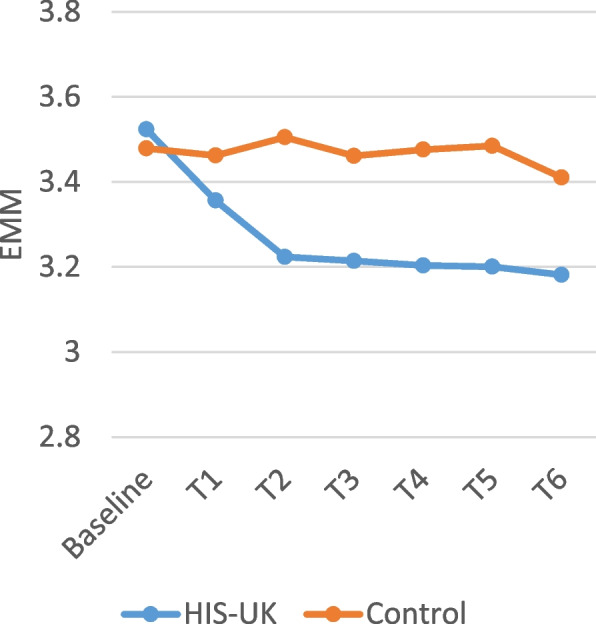
Condom use experience scale estimated marginal means by trial arm and time

**Fig. 3:**
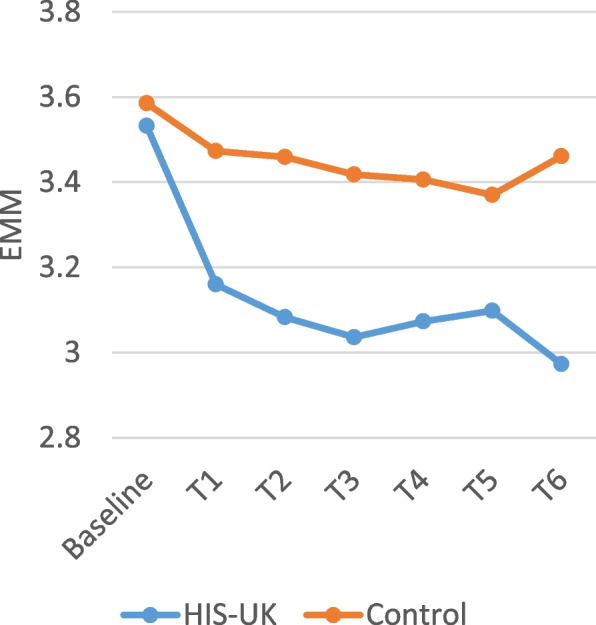
Condom use attitudes scale estimated marginal means by trial arm and time

**Fig. 4:**
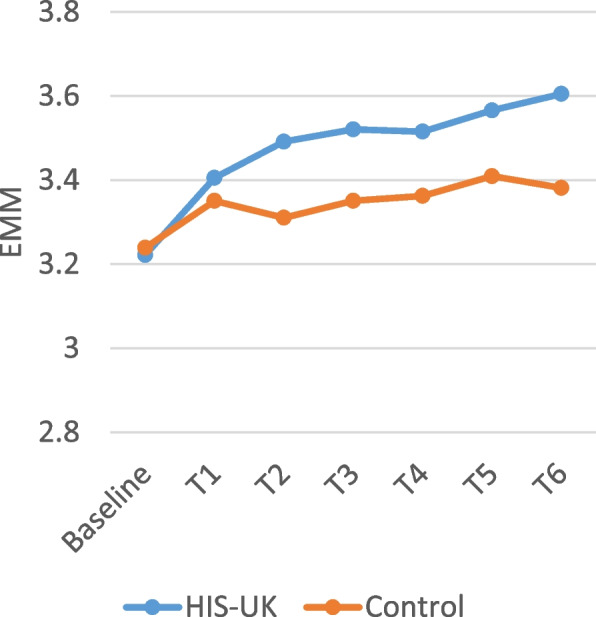
Condom use self-efficacy 7 item scale estimated marginal means (EMM) by trial arm and time

**Fig. 5:**
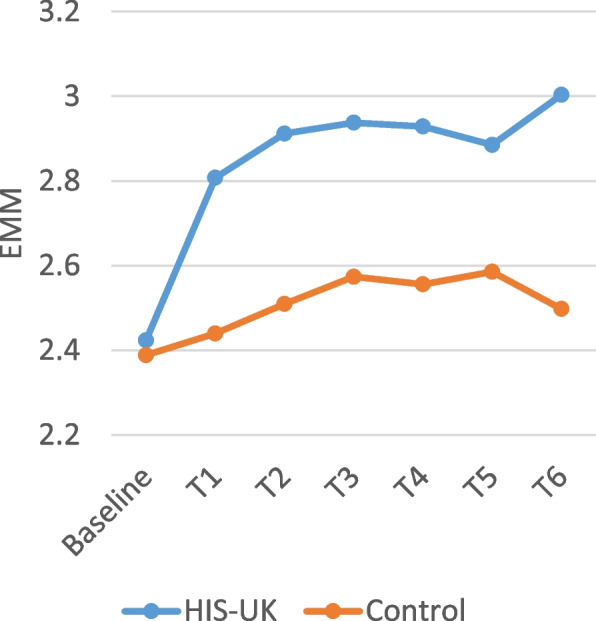
Pleasurable condom use scale estimated marginal means (EMM) by trial arm and time

**Fig. 6:**
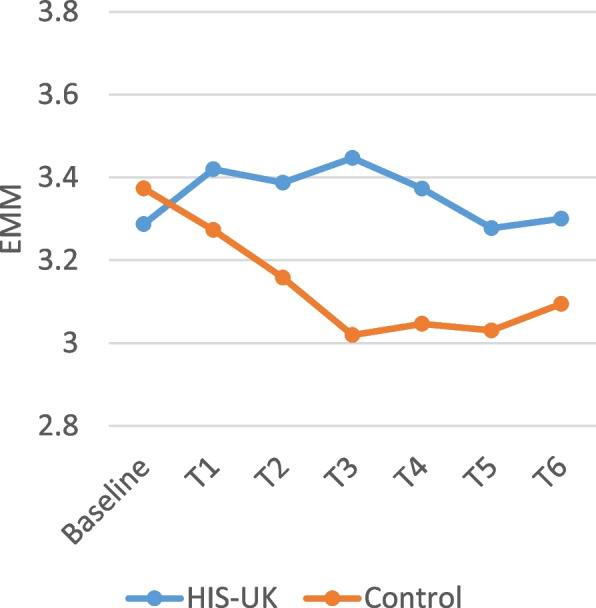
Condom use motivation scale estimated marginal means (EMM) by trial arm and time

Table 4 displays the intervention arm and the intervention arm by time interaction fixed effects derived from the linear mixed effects models for the attitudinal scale items measured by the monthly questionnaires. As shown, a significant interaction effect between the intervention arm and time was observed for all the scale items; consequently, the estimated marginal means (EMM) by trial arm and time displayed in Figs. 2, 3, 4, 5 and 6 should be considered.

**Table 4:** Tests of fixed effects for continuous secondary attitudinal scale outcomes^a^

Outcome Variable	*N*	Trial arm	Trial arm by Time
Condom use experience scale *7 item; Likert; mean score (min 1*,* max 5)* *Higher score – more barriers*	575	F = 12.8, df = 1, *p* < .001	F = 8.4, df = 6, *p* < .001
Condom use attitudes scale *5 item; Likert; mean score (min 1*,* max 5)* *Higher score – more negative attitudes*	575	F = 26.1, df = 1, *p* < .001	F = 7.6, df = 6, *p* < .001
Pleasurable condom use scale *5 item; Likert; mean score (min 1, max 5)* *Higher score – more positive*	575	F = 27.7, df = 1, *p* < .001	F = 9.1, df = 6, *p* < .001
Condom use self-efficacy scale *Likert; mean score (min 1, max 5)* *Higher score – greater self-efficacy*			
7 item	575	F = 4.4, df = 1, *p* = .035	F = 2.7, df = 6, *p* = .014
3 item	575	F = 8.5, df = 1, *p* = .004	F = 5.2, df = 6, *p* < .001
Condom use motivation scale *Single item; Likert; min 1 max 5* *Higher score – greater desire*	575	F = 5.1, df = 1, *p* = .025	F = 3.7, df = 6, *p* = .001

^a^Linear Mixed-Effects Model for repeated measures with unbalanced responses. Factors included: Trial group, Ethnicity, Site, Sexual risk, Time, Time x Trial Group

A higher score on the *Condom Use Experience Scale* and the *Condom Use Attitudes Scale* indicates greater barriers and more negative attitudes. As illustrated in Figs. 2 and 3, the estimated marginal mean (EMM) scores for both scale items indicate a significant and enduring decrease among HIS-UK participants following the delivery of the intervention post-baseline. In comparison, little change was observed in the EMM scale scores post-intervention delivery among the Control.

A higher score on the self-efficacy scales reflects greater condom confidence. As shown in Fig. 4, HIS-UK participants reported increased and sustained confidence in their ability to use condoms effectively post-baseline and intervention arm delivery (T1 through T6), after controlling for all other factors, as compared to the Control.

Similarly, compared to the Control, a marked and statistically significant impact of the HIS-UK intervention over time was observed for responses to the *Pleasurable Condom Use Scale* and *Condom Motivation Scale*. Participants expressed greater and sustained agreement that condoms can increase enjoyment of sexual experiences, following delivery of HIS-UK (Fig. 5). Furthermore, HIS-UK participants’ desire to use condoms with their sexual partners remained consistent during the follow-up period (T1-T6), whilst motivation declined among the Control (Fig. 6).

#### Behavioural

The impact of the HIS-UK intervention on the behavioural measures was more limited, with fewer effects observed.

Table 5 displays the intervention effect for the binary behavioural outcome measures, expressed as the adjusted odds ratios from the repeated measures logistic models fitted. Where a significant interaction effect between the intervention arm and time was observed, the Wald Chi-Square test statistic is presented.

**Table 5:** Intervention parameter estimates (adjusted odds ratio and Wald Chi-Square) for binary behavioural outcomes occurring in the previous 4 weeks^a^

Outcome Variable	*N*	Estimated Intervention Effect HIS-UK vs. C-group (95% CI)^1^	Time *P*-Value^2^	Trial arm by Time^2^
Condom use	532			X^2^ = 27.8, df = 6, *p* < .001
Condom use at last sex with regular partner	192	1.378 (0.820, 2.315), *p* = .225	0.445	-
Condom use at last sex with casual partner	248	1.208 (0.836, 1.747), *p* = .314	< 0.001	-
Consistency of condom use with casual partners (always vs. irregular)	237	1.198 (0.663, 2.167), *p* = .549	< 0.001	-
Correct use (coverage start to finish) for last condom used	308	1.211 (0.796, 1.844), *p* = .371	0.879	-
Condom fit and feel issues for last condom used	311	0.725 (0.470, 1.120), *p* = .148	< 0.001	-
Lubricant use	313			χ^2^ = 12.6, df = 6, *p* = .051
Lubricant use with last condom used	312	1.511 (0.967, 2.361), *p* = .070	0.281	-

^a^Generalised Estimating Equation model for a repeated measures logistic regression with factors: Trial group, Ethnicity, Site, Sexual risk, Time, Time x Trial Group; ^1^Adjusted Odds Ratio of model parameter estimates; ^2^Wald Chi-Square

**Fig. 7:**
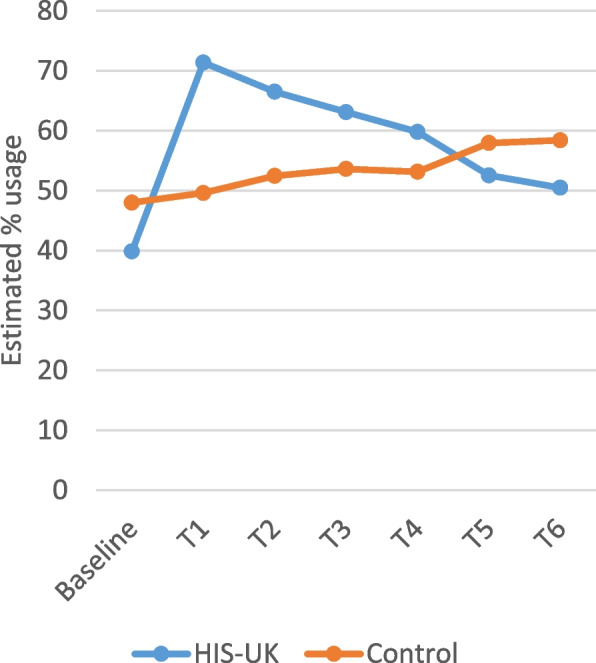
Estimated percent usage of condoms during last month by trial arm and time

A strong interaction effect of the intervention arm with time for usage of condoms with sexual partners during the previous 4-week period was observed (Wald χ^2^ = 27.8, df = 6, *p* < .001). Displayed in Fig. 7, in the first month post-intervention, condom use among sexually active participants in the HIS-UK arm increased, with a heightened likelihood of condom use during the month compared to the Control. This effect diminished, however, with time and usage was at a similar level between the groups beyond T4.

No apparent intervention effect or time by intervention interaction was evident for condom use at last sex by partner type. Furthermore, no intervention effect was seen when consistency of condom use with casual partners, as measured by the binary outcome of irregular condom use during the month versus usage at every act, was modelled (AOR: 1.198 (95% CI: 0.663, 2.167), *p* = .549). However, there was a significant time effect for the behavioural outcomes of condom use at last sex with a casual partner, and consistency of condom use with casual partners (see Table 5); condom use and consistency of use was more likely at every month post intervention as compared to baseline (reference category).

**Fig. 8:**
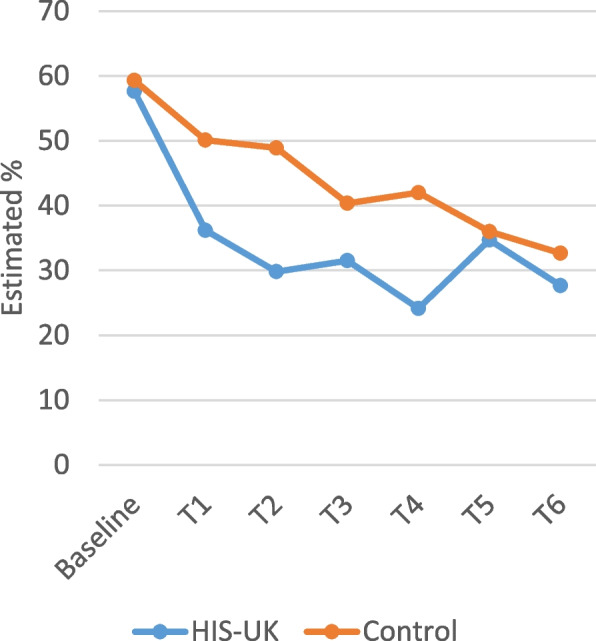
Estimated percent reporting poor fit and feel of last condom used by trial arm and time

Figure 8 shows there was also a significant effect of time (Wald χ^2^ = 27.88, df = 6, *p* < .001, data not shown in Table 5) on condom comfort. The model revealed a consistent decline in the predicted percentage of participants reporting poor fit and feel of their last condom from baseline to T6. There was no apparent effect of HIS-UK (AOR: 0.725 (95%CI: 0.470, 1.120), *p* = .148), as the decline was evident in both trial arms.

The likelihood of lubricant usage at monthly intervals post intervention delivery was greater among the HIS-UK participants than the Control during the months following intervention delivery (Wald χ^2^ = 12.55, df = 6, *p* = .051). Furthermore, the odds of lubricant usage at last condom usage were 51% higher among HIS-UK participants than the Control (AOR: 1.511 (95% CI: 0.967, 2.361), *p* = .070),

Table 6 displays the intervention arm EMM score difference from the linear mixed-effect models fitted for the two behavioural score measures of condom use: errors and problems and condom fit and feel models fitted.

**Table 6:** Estimated effect (EMM difference) of the HIS-UK intervention and tests of fixed effects for the behavioural score outcomes^a^

Outcome Variable	*N*	Estimated Intervention Effect HIS-UK vs. Control (95% CI)^1^	Trial arm by Time
Condom use errors and problems score *Higher score – more errors*	537	− 0.459 (−0.886, − 0.033), *p* = .035	F = 2.8, df = 6, *p* = .012
Condom fit and feel score *Higher score – poorer fit and feel*	537	− 0.095 (−0.300, 0.110), *p* = .363	F = 1.9, df = 6, *p* = .082

^a^Linear Mixed-Effects Model for repeated measures with unbalanced responses; Factors included: Trial group, Ethnicity, Site, Sexual risk, Time, Time x Trial Group. ^1^Pairwise comparison based on estimated marginal means

**Fig. 9:**
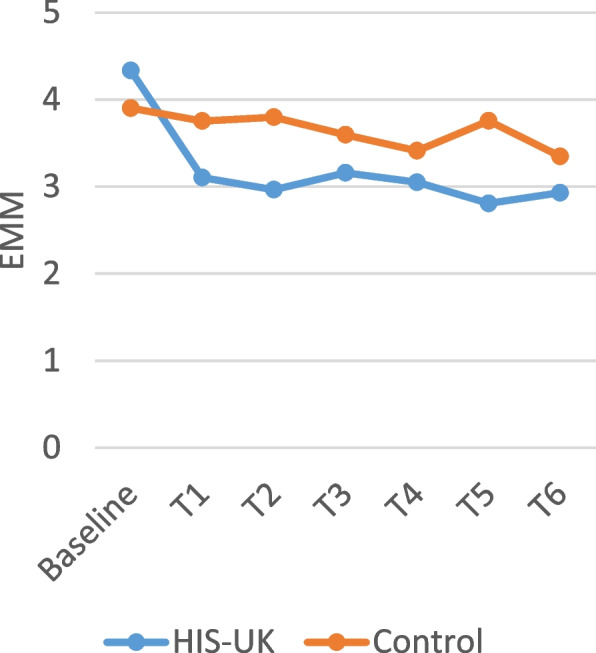
Condom use errors and problems score estimated marginal means by trial arm and time

After adjusting for ethnicity, recruitment site, and sexual risk, the average condom use error score was significantly lower among the HIS-UK participants than the Control (EMM diff: − 0.459, 95% CI − 0.886, − 0.033, *p* = .035), with a clear intervention by time interaction (F = 2.76, df = 6, *p* = .012). Figure 9 illustrates a marked and consistent decline in condom use errors after baseline among the HIS-UK participants. An intervention-by-time interaction trend was also observed for the fit and feel scores of condoms used during the last month, (F = 1.88, df = 6, *p* = .082). Reported problems with condom fit-and-feel remained evident in the few months post-baseline among the control participants prior to declining. In comparison, the HIS-UK participants reported improved condom fit-and-feel following intervention delivery, sustained up to five months post-delivery (Figure not shown).

## Discussion

The aim of this trial was to evaluate the effectiveness of the HIS-UK intervention to reduce chlamydia test positivity through the promotion of correct and consistent male condom use. Following IMBS principles for behaviour change, the HIS-UK intervention was designed to equip individuals with the essential information, skills and necessary resources to empower them to make informed decisions regarding their sexual behaviour and condom use, whilst improving condom positivity and condom use self-efficacy [36–38]. In doing so, the intervention sought to challenge and dispel negative experiences and perceptions surrounding condom use, thereby promoting healthier choices and practices for STI prevention.

One of the major barriers to condom use is known to be negative attitudes. These attitudes can encompass a range of sentiments, such as reluctance, discomfort, or resistance and may stem from various factors, including cultural, social or interpersonal influences and past experiences. Addressing poor condom use attitudes is therefore essential for promoting safe sexual practices and reducing the risk of STIs [39–41]. The HIS-UK intervention has demonstrated its ability to have a robust and sustained positive effect on condom-related beliefs and perceptions. Post-intervention delivery, HIS-UK participants had significantly more positive perceptions of condoms and identified fewer barriers to their use than control participants. Additionally, they expressed a greater level of agreement that condoms can be enjoyable and are able to enhance sexual experience and pleasure. Participants who underwent the intervention also reported increased confidence in using condoms correctly and effectively. Moreover, and most importantly, they expressed a continued desire to use condoms with their sexual partners.

At baseline, 41% of participants reported use of a condom during the previous month. Post intervention delivery, sexually active HIS-UK participants displayed a noteworthy increase in the likelihood of condom use compared to control participants, which gradually declined during follow-up. This highlights the effectiveness of HIS-UK in promoting safer sexual practices that have the potential to extend beyond the immediate post-intervention outcome and underscores the importance of conducting longitudinal assessments to understand fully the impact of sexual health interventions in the short, medium and longer-term. This is particularly pertinent given that the chlamydia transmission probability for each unprotected sex act is estimated to be in the range of 0.10–0.18, and per-partnership, rates of 30–55% are seen [42–45].

The correct and consistent use of condoms is crucial for effective prevention of direct skin-to-skin contact and the exchange of bodily fluids for protection against STIs [46, 47]. Findings in this trial showed consistency of condom use with casual or new partners improved post-baseline among both the control and HIS-UK participants. The absence of a discernible intervention effect raises questions about the precise mechanism of the HIS-UK intervention in influencing condom usage during potentially riskier encounters; it would appear that receiving any professionally delivered condom advice and care improved consistent usage within this sexual context. Indeed, the control participants in the trial received usual NHS information and care, which typically included access or signposting to free condom supplies.

Correct condom use at last usage was assessed with two measures: (a) complete coverage, denoting the use of a condom from start to finish of sex, and (b) the Condom Use Errors and Problems Score. The odds of achieving complete condom coverage at last usage were greater among HIS-UK participants in comparison to control participants; however, this was not statistically significant.

Condom failure can occur for various reasons, including incorrect use (such as improper placement, unrolling, or removal), condom damage from improper handling or sexual activity, and slippage or leakage from poor fit. Examination of monthly reported condom use errors and problems showed a significant intervention effect favouring the HIS-UK arm. Participants who received the HIS-UK intervention reported fewer errors and problems than control participants. Notably, a significant intervention by time interaction effect was also observed, indicating longevity of impact.

Issues with the fit-and-feel of condoms are commonly cited by men who report inconsistent or incorrect condom use, along with application problems, reduced sensation and erection difficulties. Such negative experiences are likely to be responsible for much of the variation in condom use self-efficacy and outcome expectancies known to be related to consistent condom use or lack thereof [15, 48–51]. HIS-UK provided a variety of condoms and lubricants to participants to explore for fit and feel in the absence of a sexual partner. This approach aimed to promote condom use and condom use self-efficacy whilst alleviating any performance-related anxieties associated with a partner’s presence. By trying out these products solo, individuals could explore a range of condom options (possibly for the first time with lube), focus on their own pleasure without external pressures, enabling them to gain familiarity with correct usage techniques and identify products most suited to them in terms of fit, feel and ease of use.

Measures of lubricant use and condom comfort (fit and feel) were used to assess the effect of the HIS-UK intervention on condom use experiences. Use of a water-based lubricant is associated with several benefits, including a reduction in condom failure rates due to reduced friction, whilst not increasing condom slippage. Lubrication also enhances the overall sexual experience by reducing irritation and discomfort and increasing sensitivity and overall pleasure. Furthermore, applying a lubricant can make it easier to put on condoms, reducing the chances of incorrect application [52]. The findings show the likelihood of lubricant use was greater among the HIS-UK participants than control participants following intervention delivery, with an associated significant intervention by time interaction.

The analysis of condom fit and feel experience is somewhat less clear. While there was a consistent decline in reporting poor fit and feel of the last condom used from baseline to T6, indicating an overall positive trend, the absence of a discernible HIS-UK program effect raises questions about the specific factors driving this decline. The intervention arm by time interaction effect (at the 10% level of significance) for general fit and feel scoring based on all condoms used during the month adds a layer of complexity, suggesting potential variability in participant recall or in the program’s impact across different measures of condom satisfaction. Further research to unravel the complexity of measuring intervention outcomes such as condom comfort in real-world scenarios is therefore warranted.

Unfortunately, due to COVID-19, the HIS-UK study was unable to fully recruit to target in the time available. As such, the study was insufficiently powered to draw firm conclusions regarding its overall impact on chlamydia test positivity at six months. Despite this limitation, a promising trend emerged as HIS-UK participants exhibited a notable 4.9% point reduction compared to the control group at six months. Although this reduction was not statistically significant, it is noteworthy and warrants consideration of the implementation of HIS-UK in the realm of public health interventions targeting the prevention of STIs.

Previous evaluations of interventions promoting condom use have often found modest effects on frequency of condom use [for reviews, see [11, 12]; however, it is difficult to compare many of our findings on our secondary outcomes such as condom use attitudes, condom use self-efficacy and condom errors and problems, as few previous trials have included these variables.

### Strengths and limitations

There were several strengths of this study, including our recruitment of a fairly diverse sample with regard to education and social deprivation, the inclusion of an STI biomarker as the primary outcome variable, and the use of psychometrically valid questionnaires. The HIS-UK trial faced significant challenges and disruption due to the COVID-19 pandemic and its legacy. To align with the trial’s original objectives, several protocol amendments were made. Many of these amendments bolstered the trial, yielding valuable insights for the conduct of sexual health research in the future. Conversely, other changes, though essential to striving toward the original goals, ultimately diminished the power of the trial to detect impact and assess effectiveness.

Specifically, the inclusion of community recruitment via PIC direct text messaging greatly boosted participant numbers; however, the chlamydia test positivity rate among the broader community population of young men was lower than that of those attending specialised services. This alteration meant that the assumptions made in the original power and sample size calculations were less relevant.

Additionally, the amendments to reflect reduced face-to-face clinical contact post COVID-19, for example online self-registration and the option for intervention delivery via telephone and video consultations, enabled the delivery of the trial to continue. However, the change meant a substantial reduction in baseline activity completion, despite the dedicated efforts of staff to follow up with participants. Previously, all recruitment and baseline activities took place during a single consultation and attrition was minimal.

We extended our STI screening to offer postal chlamydia screening kits to participants, which proved popular. However, a significant proportion of participants ordered kits but failed to return samples, despite follow-up reminders. Had all samples been collected in clinical settings as initially proposed, we believe our screening completion rates would have been higher.

Finally, participants were required to complete monthly surveys and a qualitative sub-study with 25 of our participants indicated that some participants found the surveys somewhat “tedious” and “repetitive”. This may have been one reason why some participants dropped out of the study [53].

## Conclusion

In summary, the HIS-UK intervention has demonstrated a substantial influence on participants’ condom-related attitudes, mitigating barriers, fostering positive perceptions, and instilling greater confidence in condom use. Moreover, the findings indicate that HIS-UK directly influenced participants’ sexual behaviours, leading to a higher likelihood of condom usage and improved self-efficacy, with fewer reported errors and problems. The sustained positive transformation over time further indicates HIS-UK’s effectiveness in shaping long-term changes in condom-related beliefs and behaviours and as such its potential to play a pivotal role in the endeavour to reduce STI rates.

## Supplementary Information

Supplementary Material 1.

## Data Availability

The datasets used and/or analysed during the current study are available from the corresponding author on reasonable request or via the University of Southampton repository: researchdata@soton.ac.uk.

## Abbreviations

AOR: Adjusted Odds Ratio
COVID-19: Coronavirus Disease 2019
CTRS: Clinical Trial Recruitment Site
DBCI: Digital Behaviour Change Interventions
EMM: Estimated Marginal Mean
GP: General Practice
GUM: Genitourinary Medicine
HIS-UK: Home-based Intervention Strategy, United Kingdom
IMBS: Information-Motivation-Behavioural Skills
NIHR: National Institute for Health and Social Care Research
NHS: National Health Service
NICE: National Institute for Health and Care Excellence
PIC: Patient Identification Centre
STI: Sexually Transmitted Infection
