# Supplementary material for: Evaluating the home-based intervention strategy (HIS-UK) to reduce new chlamydia infection among young men aged 16–25 years by promoting correct and consistent condom use: findings from a randomised controlled trial
Source: BMC Health Serv Res. 2024 Dec 18;24:1607. doi: 10.1186/s12913-024-11911-2 (PMC11654348; doi:10.1186/s12913-024-11911-2)
Supplement: Supplementary file 1 — Supplementary Material 1. [file 12913_2024_11911_MOESM1_ESM.pdf]

Background Information

D_1

Please answer the following questions about yourself

How old are you? [-]

What is your ethnic group?
Choose the one option that best describes your ethnic group or background

- ○ White British
- ○ White Irish
- ○ White Other
- ○ Black Caribbean
- ○ Black African
- ○ Black Other
- ○ Chinese
- ○ Japanese
- ○ Pakistani
- ○ Indian
- ○ Bangladeshi
- ○ Asian Other
- ○ Mixed - White and Black Caribbean
- ○ Mixed - White and Black African
- ○ Mixed - White and South Asian
- ○ Mixed - Other
- ○ Any other ethnic group

What is the highest level of education you have completed? [Please select]

Are you in education/training? [Please select]

Are you in paid employment? [Please select]

What is your postcode? [ ]

General Health

H_1

Please answer the following questions about your health

How would you rate your health in general? | Please select

How would you rate your health TODAY?
Click along the bar to move the slider indicator

Worse health you can imagine — Best health you can imagine

Does your health TODAY, give you problems...

- Doing usual activities? (work, study, family or leisure) | Please select
- Walking about? | Please select
- Washing and dressing yourself? | Please select

Does your health TODAY, limit you from...

- Doing moderate activities? (carrying a chair, pushing a bike) | Please select
- Climbing several flights of stairs? | Please select

In the LAST 4 WEEKS have you...

- Accomplished less than you would like because of your physical health? yes ○ no ○
- Been limited in the kind of work or other activities you could do because of your physical health? yes ○ no ○

General Health

H_2

Please answer the following questions about how you feel
If you are unsure how to answer a question, please give the best answer you can

Which of the following best describes any pain or discomfort you feel TODAY? | Please select

In the LAST 4 WEEKS how much did pain interfere with your normal activities? | Please select

Next

General Health

H_3

Please answer the following questions about how you feel emotionally

Which of the following best describes your emotional health TODAY? Please select

In the LAST 4 WEEKS have you...

Accomplished less than you would like as a result of any emotional problems (such as feeling depressed or anxious)? yes ○ no ○

Done work or activities less carefully than usual as a result of any emotional problems? yes ○ no ○

Next

General Health

H_4

For each question, please give the one answer that is closest to the way you have been feeling

In the LAST 4 WEEKS how much of the time...	All the time	Most of the time	Some of the time	A little of the time	None of the time
Have you felt calm and peaceful?	○	○	○	○	○
Did you have a lot of energy?	○	○	○	○	○
Have you felt down-hearted and blue?	○	○	○	○	○
Has your physical or emotional health interefered with your social activities (like seeing friends)?	○	○	○	○	○

Next

Medication

H_5

Please answer the following questions about any medications you take, or have taken in the past

Have you ever used, or are currently taking, any of the following?
Please tick any that apply or leave blank

- ☐ Blood pressure medication
- ☐ Cardiac medicines (e.g. Digoxin, Beta blockers)
- ☐ Psychiatric medicines (e.g. Fluoxetine, Opiods)

Have you ever taken, or do you take, medications to improve your erection?
e.g. Viagra, Sildenafil, Cialis

yes ○ no ○

Next

Use of Health Services

HE_1

Please answer the following questions about your use of health services for your sexual health and contraceptive needs

In the LAST 4 WEEKS have you visited or spoken to any services about your sexual health or contraceptive needs? yes ○ no ○

If yes, which services?
Please tick all that apply

- ☐ Sexual health, contraception or GUM service
- ☐ GP surgery / Health centre
- ☐ NHS walk-in centre
- ☐ GP out of hours service
- ☐ NHS outpatients
- ☐ NHS 111 / NHS Direct Call phone service
- ☐ Pharmacy consultation
- ☐ Minor Injuries / AE Department
- ☐ Work, school or college nurse / welfare officer / counsellor / support worker

Next

Use of Health Services

HE_1b

Please answer the following questions about your use of health services for your sexual health and contraceptive needs

How many face-to-face (in-clinic) or telephone/video contacts you have had in the LAST 4 WEEKS

	In-clinic consultation	Telephone/video consultation
Sexual health, contraception or GUM service	-	-
GP surgery / Health Centre: Doctor	-	-
GP surgery / Health Centre: Nurse	-	-
NHS walk-in Centre	-	
GP out of hours service	-	-
Outpatients	-	-
NHS 111 / NHS Direct Call		-
Pharmacy consultation	-	-
Minor Injuries / A&E department	-	
Work / School / College: Nurse	-	-
Welfare officer, counsellor or support worker	-	-

Next

Use of Health Services

HE_1c

Please answer the following questions about your attendance at sexual health, contraception and GUM services ONLY

Thinking ONLY about when you attended sexual health, contraception or GUM services in the LAST 4 WEEKS...

Did you need to take time off paid work? yes ○ no ○

IF YES, how many hours did you take off? [0 ▾]
Please give total for all visits to the services in last 4 weeks

Did you need to take time away from education or training? yes ○ no ○

IF YES, how many hours did you take off? [0 ▾]
Please give total for all visits to the services in last 4 weeks

Were you accompanied by a partner, friend or relative? yes ○ no ○

IF YES, how many hours did they accompany you for? [0 ▾]
Please give total for all visits to the services in last 4 weeks

[- ▾]

IF YES, what would that person/people have been doing if they had not been with you?

- ○ Paid work
- ○ Attending education
- ○ Leisure activities
- ○ Other

Next

HIS UK

Use of Health Services

HE_1d

Please answer the following questions about your attendance at sexual health, contraception and GUM services

When you attended sexual health, contraception or GUM services in the LAST 4 WEEKS...

How did you travel to the service?
If you attended more than once please give the answer which applies to most of your visits

I walked	○
By car	○
By public transport (bus, train)	○
By taxi	○

What was the cost of any fares or parking fees for each return journey (there and back)?

[0] Pounds [0] Pence

Please estimate how long each return journey took (there and back)?

[0] Hours [0] Mins

Next

Use of Health Services

HE_2

Please answer the following questions about your use of health services for your sexual health and contraceptive needs

In the LAST 4 WEEKS how many times have you...

Ordered a free online STI testing kit?	-
Ordered free condoms online?	-
Obtained free condoms from a clinic or service?	-

In the LAST 4 WEEKS have you received any of the following from a clinic or service?
Please tick any that apply or leave blank

- ☐ Hepatitis B vaccination
- ☐ Hepatitis B immunogobulin
- ☐ PrEP (HIV pre-exposure prophylaxis)
- ☐ PEP/PEPSE (HIV post-exposure prophylaxis)
- ☐ Antibiotics to treat an STI
- ☐ HIV antiretroviral medication

Have you stayed in an NHS hospital overnight because of a sexual health related problem in the last 4 weeks? yes ○ no ○

IF YES, for how many nights? 0

Next

Sexual Health Costs

HE_3

Please answer the following questions about your expenditure on treatment and products for your sexual health and contraceptive needs

In the LAST 4 WEEKS how much have you spent on the following...

	Pounds (£)
Condoms	-
Lubricants (lube)	-
STI/HIV testing kits	-
PrEP	-

Next

Background Information

D_2

Please answer the following questions about your sexual history

When we use the term **SEX** we include **vaginal intercourse and anal intercourse** when the penis enters the vagina or anus

Who have you ever had sex with?
Please tick all that apply

- ☐ Women
- ☐ Men
- ☐ Non-Binary / Other

How many sexual partners have you had in your lifetime?
Someone you have had vaginal and/or anal intercourse with

[- ▾]

Have you ever been diagnosed with a sexually transmitted infection (STI)? yes ○ no ○

If yes, please tick all that apply

- ☐ Chlamydia
- ☐ Gonorrhoea
- ☐ Genital Warts
- ☐ Genital Herpes
- ☐ Syphilis
- ☐ Other genital infection

Have you been diagnosed as HIV positive? yes ○ no ○

To your knowledge, have you ever made anyone pregnant? yes ○ no ○

Background Information

D_2a1

Please answer the following questions about your current relationship

Are you in a sexual relationship?<br>Having vaginal and/or anal intercourse with a regular/long term partner	yes ○	no ○
Do you live with your partner?	yes ○	no ○
Are you having sex with other people whilst in this relationship?	yes ○	no ○

Next

Background Information

D_2a2

Please answer the following question about your sexual activity

CASUAL SEX PARTNERS include friends with benefits, sex buddies, booty calls and one-night stands

Which of the following statements best describes your current sexual activity?

- ○ I have frequent casual sex
- ○ I have occasional casual sex
- ○ I am currently not sexually active

Next

Condoms & Lubes

D_3

This section asks about your use of condoms and lubricants (lubes)

Have you ever seen a condom demonstration or been taught how to use a condom correctly? yes ○ no ○
By a professional (e.g. teacher/youth worker/doctor/nurse)

Have you ever used a condom during sex? yes ○ no ○
During vaginal or anal intercourse

Have you ever used an additional lube during sex...

a) with a condom? yes ○ no ○

b) without a condom? yes ○ no ○

Next

Recent Sexual Behaviour

T0_1

The following section ask about your sex life during the LAST 4 WEEKS

Have you had sex in the last 4 weeks?
Vaginal and/or anal intercourse

yes ○ no ○

Next

Recent Sexual Behaviour

T0_1a

We would like to ask about your sexual behaviour in the LAST 4 WEEKS
If you cannot give specific numbers please enter your best estimate.

In the **LAST 4 WEEKS**...

How many people have you had sex with? [-]
Vaginal or anal intercourse

Who did you have sex with?
Please tick all that apply

- ☐ Women
- ☐ Men
- ☐ Non-Binary / Other

Did you have sex with a casual or new partner? yes ○ no ○
Casual partners include friends with benefits, sex buddies, booty calls and one-night stands

Did you have sex with a long-term / regular partner? yes ○ no ○

Did you use a condom during sex in the last 4 weeks? yes ○ no ○

Next

Condom Use

T0_2

This section asks a bit more about why you have not used condoms recently

In the **LAST 4 WEEKS**...

How many times have you had sex?
Vagina l and/or anal intercourse

-

Why have you not used condoms?
Please tick all reasons that apply

- ☐ Didn't have any available
- ☐ Don't like using them
- ☐ Used other forms of contraception
- ☐ Partner refused / didn't want to use them
- ☐ Partner(s) and I are clear of STIs
- ☐ Condom broke
- ☐ Trying to get pregnant
- ☐ Didn't think about it
- ☐ Didn't feel I could ask
- ☐ Taking PrEP

If there are other reasons please list them here:

Next

Condom Use

T0_3

This section asks about your recent use of condoms with CASUAL or NEW

CASUAL SEX PARTNERS include friends with benefits, sex buddies, booty calls and one-night stands

In the LAST 4 WEEKS, thinking about the sex you had with any casual or new partners...

How many times did you have sex?
Vaginal and/or anal intercourse

-

How frequently did you use condoms during VAGINAL sex?

Please select

How frequently did you use condoms during ANAL sex?

Please select

Did you use a condom the LAST time you had sex with a casual or new partner?

yes ○ no ○

Next

Condom Use

T0_3a

This section asks about your recent use of condoms with your LONG-TERM or REGULAR partner(s)

In the LAST 4 WEEKS, thinking about the sex you had with any long-term or regular partners...

How many times did you have sex? [-]
Vaginal and/or anal intercourse

How frequently did you use condoms during VAGINAL sex? [Please select]

How frequently did you use condoms during ANAL sex? [Please select]

Did you use a condom the LAST time you had sex with a long-term or regular partner? yes ○ no ○

Next

This section asks a bit more about your recent use of condoms

In the LAST 4 WEEKS, why did you use condoms?
Please tick all that apply

- ☐ to avoid sexual transmitted infections
- ☐ to avoid HIV/AIDS
- ☐ to please my partner
- ☐ to make sex better
- ☐ to make sex last longer
- ☐ to avoid making a mess
- ☐ for fun / pleasure
- ☐ to avoid getting my partner pregnant

If there are other reasons please list them here:

Where did you get the condoms you used in the LAST 4 WEEKS?
Please tick all that apply

- ☐ Free from a health service or worker (clinic/doctor/nurse/youth worker/c-card)
- ☐ Free on-line ordering service
- ☐ Bought from a shop, pharmacy or online
- ☐ Partner provided them
- ☐ From a friend

Lubricant Use

T0_3c

This section asks a bit more about your recent use of lubricants (lube)

Did you use any extra lube with any of the condoms you used in the LAST 4 WEEKS? yes ○ no ○

Did you use any extra lube the LAST time you used a condom? yes ○ no ○

Next

HIS UK

Condom Use

T0_4

The following questions asks you to think about the LAST TIME you used a condom

The **LAST TIME** you used a condom for sex (vaginal or anal intercourse) did you...

Check the expiry date?	yes ○	no ○	don't know / unsure ○
Check the condom for visible damage before having sex?	yes ○	no ○	don't know / unsure ○
Put it on the wrong side up and have to flip it over?	yes ○	no ○	don't know / unsure ○
Squeeze the air out of the tip before putting it on?	yes ○	no ○	don't know / unsure ○
Leave a space at the tip of the condom when putting it on?	yes ○	no ○	don't know / unsure ○
Lose or start to lose your erection while putting it on?	yes ○	no ○	don't know / unsure ○
Use an oil-based lubricant (like baby oil, massage oil or Vaseline) with the condom?	yes ○	no ○	don't know / unsure ○
Lose or start to lose your erection during sex while using the condom?	yes ○	no ○	don't know / unsure ○
Let the condom contact anything sharp (jewellery/teeth/piercings) before or during sex?	yes ○	no ○	don't know / unsure ○

Next

HIS
UK

Condom Use

T0_5

CONTINUED...

The **LAST TIME** you used a condom for sex (vaginal or anal intercourse) did you...

Start having sex (let your penis enter the vagina or anus) without the condom, then put it on later?	yes ○	no ○	don't know / unsure ○
Take the condom off during sex and continued without using one?	yes ○	no ○	don't know / unsure ○
Have the condom break during sex?	yes ○	no ○	don't know / unsure ○
Have the condom slip off during sex?	yes ○	no ○	don't know / unsure ○
Have the condom slip off you as you were pulling out from your partner?	yes ○	no ○	don't know / unsure ○
Have any problems with the way the condom fitted you?	yes ○	no ○	don't know / unsure ○
Have any problems with the way the condom felt?	yes ○	no ○	don't know / unsure ○

Next

Condom Fit & Feel

T0_6

Whilst thinking about the condoms you have used say how much you agree or disagree with the following statements about fit and feel

The condoms I use ...	Strongly disagree	Disagree	Neither agree nor disagree	Agree	Strongly agree
Are too long for my penis<br>Unrolled condom left at the base of my penis	○	○	○	○	○
Are too short for my penis<br>Do not cover my penis completely	○	○	○	○	○
Are too tight for my penis	○	○	○	○	○
Are too loose for my penis	○	○	○	○	○
	Strongly disagree	Disagree	Neither agree nor disagree	Agree	Strongly agree

Next

Views on Condoms

T0_7

Indicate whether you agree or disagree with the following statements about condoms
There are no right or wrong responses

	Strongly disagree	Disagree	Neither agree nor disagree	Agree	Strongly agree
Using a condom can be exciting	○	○	○	○	○
Condoms rub and cause irritation	○	○	○	○	○
Condoms interrupt the mood	○	○	○	○	○
Condoms are fun	○	○	○	○	○
Condoms can make sex last longer	○	○	○	○	○
You feel closer to your partner without a condom	○	○	○	○	○
Condoms don't feel good	○	○	○	○	○
	Strongly disagree	Disagree	Neither agree nor disagree	Agree	Strongly agree

Next

Views on Condoms

T0_7a

CONTINUED...

	Strongly disagree	Disagree	Neither agree nor disagree	Agree	Strongly agree
Condoms don't fit right	○	○	○	○	○
Condoms can help you have better sex	○	○	○	○	○
Condoms reduce orgasm/climax	○	○	○	○	○
Condoms can make sex more pleasurable	○	○	○	○	○
Condoms can add excitement to foreplay	○	○	○	○	○
Condoms feel unnatural	○	○	○	○	○
Condoms can feel good for both partners	○	○	○	○	○
	Strongly disagree	Disagree	Neither agree nor disagree	Agree	Strongly agree

Next

Condoms Use Skills

T0_8

On a scale of 1 (very difficult) to 5 (very easy) please respond to the following statements about condoms

How easy or difficult would it be for you to ...

	Very difficult 1	2	3	4	Very easy 5
Find condoms that fit you properly?	○	○	○	○	○
Apply condoms correctly?	○	○	○	○	○
Keep a condom from drying out during sex?	○	○	○	○	○
Keep a condom from breaking during sex?	○	○	○	○	○
Keep an erection while using a condom?	○	○	○	○	○
Withdraw without the condom slipping off?	○	○	○	○	○
Wear a condom from start to finish of sex?	○	○	○	○	○
	Very difficult				Very easy

Next

Condoms Use Skills

T0_8b

Indicate how much you agree or disagree with the following statements

I feel confident ...	Strongly disagree	Disagree	Neither agree nor disagree	Agree	Strongly agree
Putting on a condom in front of a partner	○	○	○	○	○
I can use a condom without 'breaking the mood'	○	○	○	○	○
I can use a condom without reducing my or my partners sexual pleasure	○	○	○	○	○

Next

Condoms Use Motivation

T0_9

Finally, record your level of agreement or disagreement to the following statement

	Strongly disagree	Disagree	Neither agree nor disagree	Agree	Strongly agree
I want to use condoms with my partner(s)	○	○	○	○	○

Submit
